# Use of Cardiac Contractility Modulation as Bridge to Transplant in an Obese Patient With Advanced Heart Failure: A Case Report

**DOI:** 10.3389/fcvm.2022.833143

**Published:** 2022-02-16

**Authors:** Daniele Masarone, Andrea Petraio, Antonio Fiorentino, Santo Dellegrottaglie, Fabio Valente, Ernesto Ammendola, Gerardo Nigro, Giuseppe Pacileo

**Affiliations:** ^1^Heart Failure Unit, Department of Cardiology, AORN dei Colli, Monaldi Hospital, Naples, Italy; ^2^Heart Transplant Unit, Department of Cardiac Surgery and Transplants, AORN dei Colli, Monaldi Hospital, Naples, Italy; ^3^Impulse Dynamics Germany, Frankfurt, Germany; ^4^Division of Cardiology, Ospedale Accreditato Villa dei Fiori, Naples, Italy; ^5^Marie-Josee and Henry R. Kravis Center for Cardiovascular Health, Zena and Michael A. Wiener Cardiovascular Institute, Icahn School of Medicine at Mount Sinai, New York, NY, United States; ^6^Department of Medical Translational Sciences, Monaldi Hospital, University of Campania “Luigi Vanvitelli,” Naples, Italy

**Keywords:** heart failure reduced ejection fraction, advanced heart failure, cardiac contractility modulation, obesity, dilated cardiomyopathy

## Abstract

Cardiac contractility modulation (CCM) is a novel device-based therapy in patients with heart failure with reduced ejection fraction (HFrEF). In randomized clinical trials and real-life studies, CCM has been shown to improve exercise tolerance and quality of life, reverse left ventricular remodeling and reduce hospitalization in patients with HFrEF. In this case report, we describe for the first time the use of CCM as a “bridge to transplant” in a young obese patient with advanced heart failure due to non-ischemic dilated cardiomyopathy. The patient had a poor quality of life and frequent heart failure-related hospitalizations despite the optimal medical therapy and, due to obesity, a suitable heart donor was unlikely to be identified in the short term and due to severe obesity risk of complications after implantation of a left ventricular assist device (LVAD) was very high.

## Introduction

Most patients with heart failure (HF) with reduced ejection fraction (HFrEF) respond well to evidence-based pharmacological treatments and enjoy a good quality of life, with a significant prolongation of life (1). However, for reasons that are as yet unexplained, up to 10% of patients do not respond to pharmacological or non-pharmacological approaches, resulting in disease progression to the most advanced stage of HF (2, 3).

The gold standard treatment option for patients with advanced HF is heart transplantation, or alternatively left ventricular assist system (LVAD) implantation as a bridge to transplant or as a destination therapy if a heart transplant is not feasible (2).

Obesity is one of the most significant factors that strongly influence the management of patients with advanced HF (4). Indeed, for patients with end-stage HFrEF, obesity has been associated with modestly reduced survival after heart transplantation (5).

Therefore, the International Society for Heart and Lung Transplantation (ISHLT) guidelines consider BMI ≥35 kg/m^2^ to be a relative contraindication to heart transplant (6).

In addition, obese patients are more vulnerable to LVAD implantation due to their increased risks of post-procedural complications. In fact, in an analysis of 17,095 INTERMACS (Interagency Registry for Mechanically Assisted Circulatory Support) registry participants who received LVADs between 2006 and 2014, obesity was associated with higher risks of infection, device malfunction/thrombosis, cardiac arrhythmias, and hospital readmissions (7).

Cardiac contractility modulation (CCM) is a new therapy for treating patients with HF that improves the performance of the failing myocardium by delivering biphasic electrical pulses during the refractory period (8).

Here, we describe a case of advanced HF in a young obese patient in which CCM was successfully used as a bridge to transplant option, highlighting the efficacy and safety of this approach in selected patients with advanced HF.

## Case Presentation

A 26-year-old male obese (height 175 cm, weight 128 Kg, body mass index 41.8 kg/m^2^, body surface area 2.39 m^2^) patient was admitted to the Heart Failure Unit of AORN dei Colli-Monaldi Hospital in August 2019 for marked dyspnea and asthenia. The clinical and demographic parameters at admission are shown in Table 1.

**Table 1:** Demographic, clinical and echocardiographic characteristic of patient at admission.

Age	26 years
Weight	128 kg
Height	175 cm
BMI (Deveraux)	41.8
BSA (Dubois)	2.39 m^2^
BP	130/80 mmHg
HR	105 b/m
LVEDD	88 mm
LVESD	71 mm
LVEDVI	123.9 ml/m^2^
LVESVI	99,1 ml/m^2^
EF (Simplon biplane)	20%
E/e'average	14
LAVI	47 ml/m^2^
PASP	60 mmHg
IVC diameter	24
IVC collapsibility index	29,1%

*BMI, body mass index; BSA, body surface area; BP, Blood pressure; HR, heart rate; LVEDD, left ventricular end-diastolic diameter; LVESD, left ventricular end-systolic diameter; LVEDVI, left ventricular end diastolic volume index; LVESI, left ventricular end systolic volume index; EF, ejection fraction; E/e' average, ratio between Peak velocity of early diastolic transmitral flow and peak velocity of early diastolic mitral septal and lateral annular motion; LAVI, left atrium volume index; PASP, Pulmonary Artery Systolic Pressure; IVC, inferior vena cava*.

**Figure 1:**
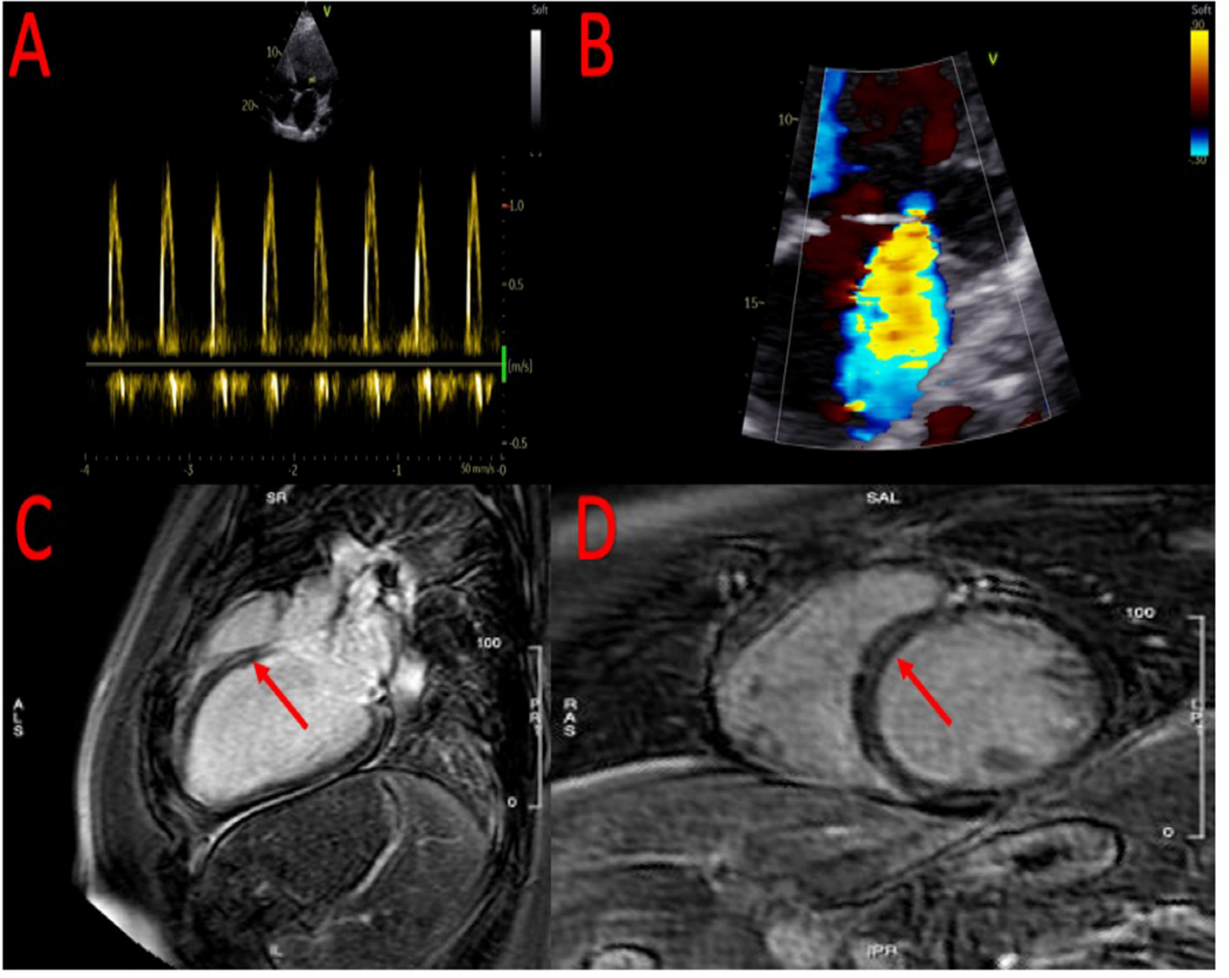
Echocardiographic evidence of severe diastolic dysfunction **(A)** and severe mitral regurgitation **(B)**. At cardiac magnetic resonance, evidence of small area of fibrosis at the basal level of the interventricular septum **(C,D)**.

After adequate therapy with diuretics and inodilators (levosimendan), which made it possible to reach a state of euvolemia and restoration of end-organ perfusion, an echocardiogram was performed with evidence of a severe reduction in left ventricular systolic function (left ventricular ejection fraction 20%), severe diastolic dysfunction and severe mitral regurgitation (Figures 1A,B; Supplementary Videos 1, 2).

To clarify the etiology of the HFrEF, coronary angiography was performed, which showed the absence of hemodynamically significant coronary lesions. Subsequently, the patient was referred for cardiac magnetic resonance imaging that confirmed the severe reduction of left ventricular ejection fraction (Supplementary Videos 3, 4) with evidence of a small area of fibrosis at the basal level of the interventricular septum (Figures 1C,D).

The patient was discharged in September 2019 and had regular outpatient check-ups in the following months to optimize therapy with the disease-modifying drugs.

As of October 2019, the patient had undergone optimized drug therapy (sacubitril/valsartan 97/103 mg bis in die, bisoprolol 10 mg once a day, eplerenone 50 mg daily), but despite this, severe left ventricular systolic dysfunction and NYHA class III persisted, and a subcutaneous ICD was implanted.

During hospitalization for ICD implantation, a cardiopulmonary test was performed to objectively evaluate his functional capacity, which indicated a severe reduction in functional capacity even after correction of the peak VO_2_ for obesity (VO_2_ peak 13.8 ml/kg/min).

Given the severe reduction in functional capacity despite optimized therapy, the patient was listed for cardiac transplantation in November 2019.

However, during follow-up, the patient experienced three episodes of acutely decompensated heart failure, requiring increased home diuretic therapy and, in one case, hospitalization for levosimendan infusion (Supplementary Figure 1).

Therefore, given the limited possibility of receiving a compatible heart within a short period, and considering the high risk of infection, device malfunction and pump thrombosis that LVAD implantation poses in obese patients, other therapeutic strategies were considered.

Mitraclip could be a viable therapeutic option for treating functional mitral regurgitation. However, the patient did not have a COAPT-like profile, and therefore this strategy would be unlikely to affect the patient's prognosis. Pulsed ambulatory infusion of levosimendan is another treatment possibility for patients with advanced HFrEF; however, this option has not been taken into account due to the severe obesity that may affect the drug efficacy.

Therefore, the only feasible therapeutic option as a bridge to transplant strategy was the off-label implantation of an Optimizer Smart^®^ (Impulse Dynamics Inc. Orangeburg, NY, USA) device to deliver CCM therapy.

The implant was performed in March 2021 (Supplementary Figure 2), and in the first 3 months after implantation, the patient showed progressive improvement in symptoms, NYHA class, quality of life (as assessed by the Minnesota Living with Heart Failure Questionnaire), echocardiographic parameters and of N-terminal brain natriuretic peptide plasma levels (Table 2).

**Table 2:** Comparison on demographic, clinical, echocardiographic and laboratory parameters between admission and 6 months follow-up.

**Parameter**	**Baseline**	**6 months follow-up**
Weight	128 kg	124
Height	175 cm	175
BMI (Deveraux)	41.8	40.4
BSA (Dubois)	2.39 m^2^	2.36 m^2^
BP	130/80 mmHg	120/70 mmHg
HR	105 b/m	88 b/m
LVEDVI	123.9 ml/m^2^	119.8 ml/m^2^
LVESVI	99.1 ml/m^2^	85.3 ml/m^2^
EF (Simplon biplane)	20%	28%
E/e'average	14	9
LAVI	47 ml/m^2^	43 ml/m^2^
PASP	60 mmHg	35
IVC diameter	24	18
IVC collapsibility index	29.1%	37.4%
NT-proBNP	3,569 pg/ml	2,256 pg/ml
MLWHFQ score	43	14

*BMI, body mass index; BSA, body surface area; BP, Blood pressure; HR, heart rate; LVEDVI, left ventricular end diastolic volume index; LVESI, left ventricular end systolic volume index; EF, ejection fraction; E/e' average, ratio between Peak velocity of early diastolic transmitral flow and peak velocity of early diastolic mitral septal and lateral annular motion; LAVI, left atrium volume index; PASP, Pulmonary Artery Systolic Pressure; IVC, inferior vena cava; NT-proBNP, N terminal pro Brian Natriuretic Peptide; MLWHFQ, Minnesota Living With Heart Failure Questionnaire*.

Six months after implantation, the patient repeated the cardiopulmonary test, which showed an increase of VO_2_ peak and a reduction in the VE/VCO_2_ ratio (Table 3).

**Table 3:** Comparison on cardiopulmonary exercise test derived data between baseline and 6 months follow-up.

**Parameter**	**Baseline**	**6 months follow-up**
RER	1.07	1.12
HR	155 b/m	166 b/m
Work	88 watts	112 watts
VO_2_ peak	13.8 ml/kg/min	16.7 ml/kg/min
Oxygen pulse (VO2 peak/HR)	8.9 ml/beat	10 ml/beat
VE/VCO_2_	34	25

*RER, respiratory exchange ratio; HR, heart rate; VCO2, carbon dioxide output; VE, ventilation; VO2, oxygen uptake*.

Through November 2021, the patient is in stable NYHA class II, following a diet to improve the possibility of a heart transplant. No further episodes of acutely decompensated heart failure occurred during follow-up.

## Discussion

Advanced HF is a clinical syndrome that is challenging to manage.

Intolerance to disease-modifying drugs is a hallmark of the end-stage phase of HFrEF, and electrical therapy such as cardiac resynchronization therapy is only indicated in patients with specific electrocardiographic characteristics (wide QRS interval, preferably with left bundle-branch block morphology), which are found in no more than 30% of all patients.

Cardiac transplantation is the gold standard therapy for these patients, and LVAD implantation is a valid treatment option, but both are limited by the presence of co-morbidities such as obesity.

In fact, a BMI >35 is related to a high risk of post-implant LVAD complications, such as drive-line infections, pump malfunction and pump thrombosis.

CCM is a new therapy for the treatment of patients with HF and severe-moderate systolic dysfunction. By delivering non-excitatory impulses to the myocardium, a series of long-term biochemical and molecular effects are induced, such as reduced expression of fetal genes (overexpressed in failing myocardium), improved calcium cycling and, finally, myocardial contraction (9, 10).

In randomized clinical trials, CCM reduces hospitalization and improves functional capacity and quality of life in patients with HFrEF (11, 12).

In a retrospective study of 68 patients who underwent CCM therapy at a follow-up after 4.5 years, the mortality of the enrolled patients was lower than predicted by the Seattle Heart Failure Model (SHFM) (13). In a retrospective single-center study, 81 patients with CCM demonstrated improvements in left ventricular ejection fraction, quality of life as measured by the Minnesota living with Heart Failure Questionnaire and a reduction in symptoms during a mean follow-up of 3 years (14). These patients had lower mortality rates than predicted by the Meta-Analysis Global Group in Chronic Heart Failure scores.

Recent results from the largest published registry to date, CCM-REG25-45, showed that the survival rates of patients with LVEF <35% were significantly higher than the survival predicted by the SHFM (*p* = 0.46) (15).

In our patient, when the advanced stage of HF was overt (documented by the frequent episodes of acutely decompensated heart failure despite optimal medical therapy), the obvious choice would have been an LVAD as a bridge to transplant, but due to the presence of severe obesity (BMI 41) the risk of complications was very high, so other therapeutic options were considered.

The MitraClip System (Abbott, Abbott Park, IL, USA), which provides transcatheter edge-to-edge repair of the mitral valve, is the most widely used device for treating functional mitral regurgitation in patients with HFrEF (16). In addition, the recent Mitrabridge registry showed that Mitraclip is a safe and effective bridge to transplant strategy for patients with advanced HFrEF (17).

However, the COAPT criteria were not met in our patient (left ventricular end-systolic diameter >70 mm, left ventricular ejection fraction ▪20%); therefore, Mitraclip implant would probably not have improved our patient's prognosis (18).

Another strategy used in patients with advanced heart failure is the periodic infusion of levosimendan (19), which can lead to an improvement in the hemodynamic profile (20) and quality of life as well as reduction of acutely decompensated heart failure episodes in patients with advanced HFrEF (21). Pulsed levosimendan infusion can be performed at fixed intervals or by assessing changes in hemodynamic parameters (22), leading to a clear clinical benefit in both cases. However, little data are available on the efficacy of this therapeutic approach in obese patients (body weight > 120 kg); therefor this therapeutic option has not been performed.

According to Federal and Drugs Administration approved physician labeling, CCM is indicated in patients with heart failure in NYHA class III-IV despite optimal medical therapy; however, no data exists regarding their use as a bridge to transplant strategy.

The above treatment strategies were thoroughly discussed in terms of expected outcomes and possible complications with the patient, who decided for CCM therapy.

Thus, in our patient for the first time, an “off-label” implant of an Optimizer Smart^®^ as a bridge to transplant strategy was performed.

During follow-up, our patient experienced a reduction in NYHA class and an improvement in quality of life, according to the Minnesota Living with Heart Failure Questionnaire. In addition, a reduced left ventricular filling pressure occurred, as documented by a reduction in the level of N-terminal prohormone of brain natriuretic peptide.

Furthermore, improvement of echocardiographic and cardiopulmonary exercise test data occurred during follow-up, indicating an increase in cardiac output (increase of VO2 peak) and a reduction of wedge pressure and pulmonary pressure (decreased VE/VCO2 slope).

## Conclusion

In this case report, we described for the first time a case of advanced HFrEF treated with CCM therapy as a bridge to transplant strategy.

Based on our case, we believe that CCM could be used as a bridge to transplant strategy in selected patients with end-stage HFrEF, not adequately compensated by pharmacological therapy with contraindications to LVAD, such as patients with severe obesity.

## Data Availability Statement

The original contributions presented in the study are included in the article/Supplementary Material, further inquiries can be directed to the corresponding author.

## Ethics Statement

Ethical review and approval was not required for the study on human participants in accordance with the local legislation and institutional requirements. The patients/participants provided their written informed consent to participate in this study. Written informed consent was obtained from the individual(s) for the publication of any potentially identifiable images or data included in this article.

## Author Contributions

DM, AP, AF, and GP: conceptualization. DM, EA, and FV: writing—original draft preparation. GN and SD: writing—review and editing. All authors have read and agreed to the published version of the manuscript.

## Conflict of Interest

AF is a full-time employee of Impulse Dynamics^®^, where Optimizer Smart^®^ was discovered and developed. The remaining authors declare that the research was conducted in the absence of any commercial or financial relationships that could be construed as a potential conflict of interest.

## Publisher's Note

All claims expressed in this article are solely those of the authors and do not necessarily represent those of their affiliated organizations, or those of the publisher, the editors and the reviewers. Any product that may be evaluated in this article, or claim that may be made by its manufacturer, is not guaranteed or endorsed by the publisher.

## Supplementary Material

Supplementary Figure 1 Timeline of the clinical case.

Supplementary Figure 2 Chest X ray showed evidence of Optimizer Smart generator (yellow line) and subcutaneous implantable cardioverter defibrillator generator (red line).
