# Supplementary figures and images for: Use of Cardiac Contractility Modulation as Bridge to Transplant in an Obese Patient With Advanced Heart Failure: A Case Report
Source: Front Cardiovasc Med. 2022 Feb 16;9:833143. doi: 10.3389/fcvm.2022.833143 (PMC8889036; doi:10.3389/fcvm.2022.833143)

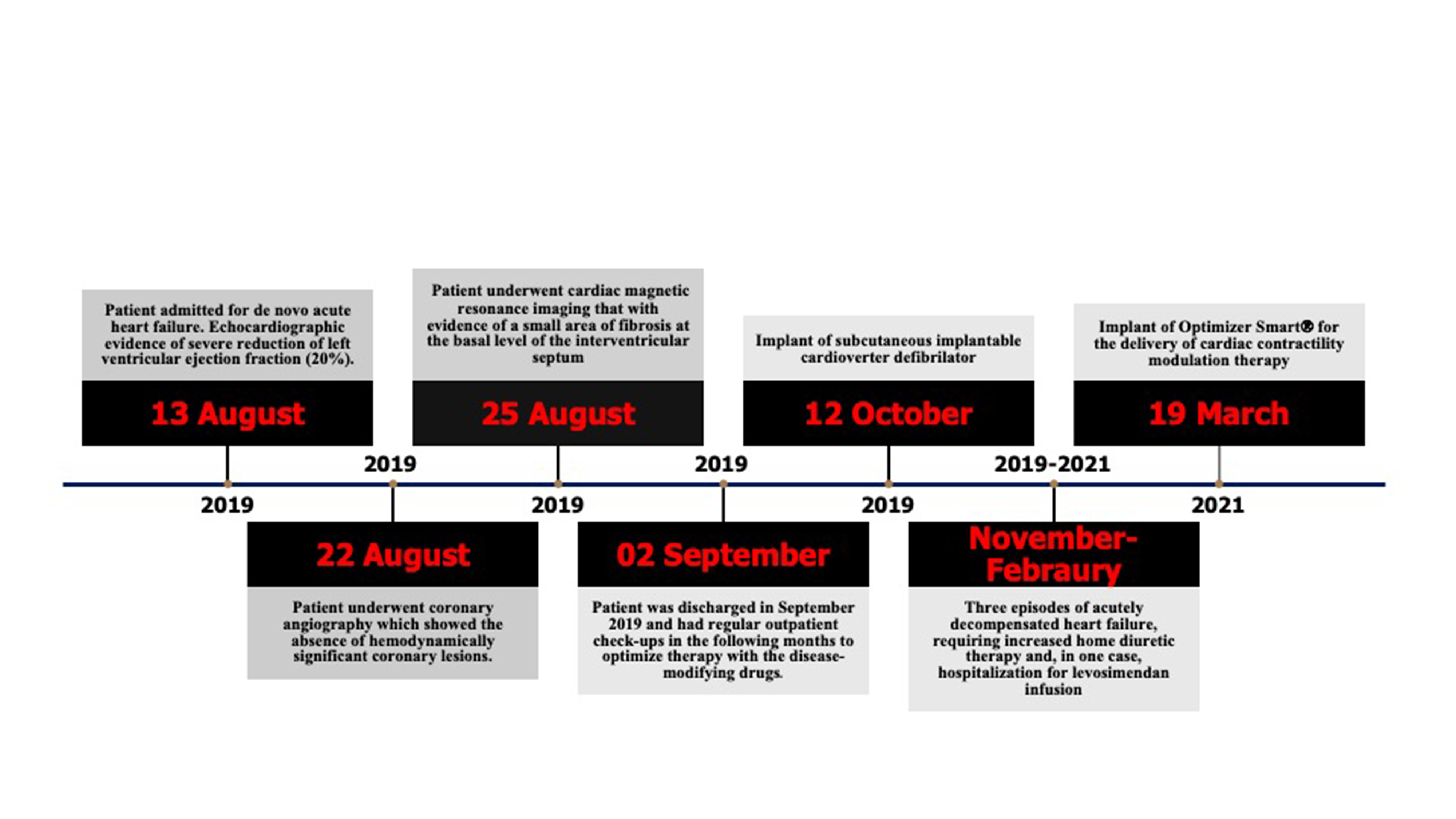

Supplement: Supplementary Figure 1 — Timeline of the clinical case. [file Image_1.JPEG]

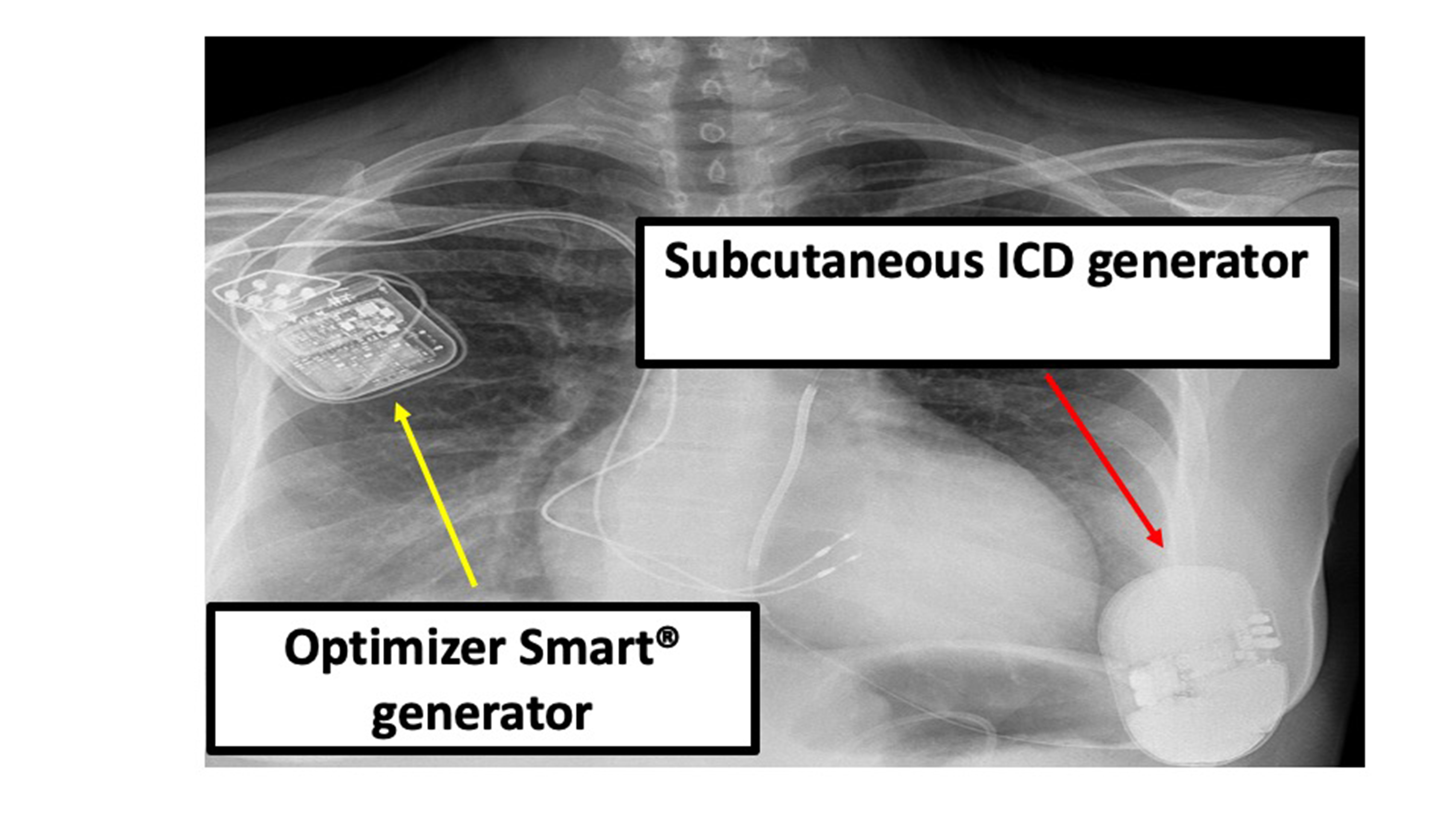

Supplement: Supplementary Figure 2 — Chest X ray showed evidence of Optimizer Smart generator (yellow line) and subcutaneous implantable cardioverter defibrillator generator (red line). [file Image_2.JPEG]
